# Effect of a plant extract of fenugreek (*Trigonella foenum-graecum*) on testosterone in blood plasma and saliva in a double blind randomized controlled intervention study

**DOI:** 10.1371/journal.pone.0310170

**Published:** 2024-09-17

**Authors:** Sindre Lee-Ødegård, Thomas E. Gundersen, Christian A. Drevon

**Affiliations:** 1 Institute of Clinical Medicine, University of Oslo, Oslo, Norway; 2 Vitas AS, Oslo Science Park, Oslo, Norway; 3 DBG AS, Oslo Science Park, Oslo, Norway; 4 Department of Nutrition, Institute of Basic Medical Sciences, University of Oslo, Oslo, Norway; University of Milan, ITALY

## Abstract

Many aging men experience reduced energy and libido related to non-optimal testosterone levels. We conducted a randomized double-blind trial with Trigozim^R^ fenugreek extract to assess impact on plasma and saliva testosterone, and some subjective effects. 95 men (40-80y) completed a 12-week intervention, taking 3 tablets daily with 0 mg (placebo; n = 22), 600 mg (n = 21), 1200 mg (n = 25) and1800 mg (n = 27) fenugreek extract and essential nutrients. Samples were collected at weeks 0, 2, 6, and 12. Participants answered a pre- and post-intervention questionnaire on lifestyle and libido. We measured total testosterone (HPLC-MS/MS) and sex hormone binding globulin (ELISA), calculated free testosterone index (FTI), and measured saliva testosterone. Plasma total testosterone and FTI increased after any dose of Trigozim^R^ vs. baseline (13.0%, p = 1.0x10^-4^ and 16.3%, p = 6.2x10^-6^), but not vs. placebo (9.0%, p = 0.122 and 11.3% p = 0.059). 1800 mg Trigozim^R^ resulted in 12.2% increased FTI (p = 0.025). Saliva testosterone concentration increased after any dose of Trigozim^R^ vs. baseline (31.1%, p = 2.3x10^-4^) and vs. placebo (37.2%, p = 0.042). 1800 mg Trigozim^R^ for 12 weeks resulted in 19.6% (p = 0.006) increased saliva testosterone. Compliance was confirmed by enhanced plasma concentration of 25-hydroxy vitamin D_3_. We observed no subjective effects or side-effects of Trigozim^R^. Trigozim^R^ increased saliva and plasma testosterone concentration during intervention but only for saliva vs. placebo. Saliva may be preferred for measuring free testosterone due to no protein-bound testosterone.

## Introduction

The active ingredient of the food supplement Trigozim^R^ is assumed to be the plant extract of fenugreek (*Trigonella foenum-graecum*), which for an extended time has been used to reduce sexual dysfunction like impotence and to enhance libido. Fenugreek is one of the oldest and most used plant extracts in traditional medicine containing substances such as steroids, alkaloids, saponins, polyphenols, flavonoids, a variety of lipids, carbohydrates, amino acids, and hydrocarbons [1].

A mixture of components of fenugreek such as fruit, stem and root has been used as spice, food, and as medicine/supplement for diabetes, high blood lipid values, obesity, various forms of cancer, inflammation, fungal and bacterial infections, and to improve libido or to have an anabolic effect during weight training [1–5].

Several studies in recent years suggest that some of the active components in fenugreek increase testosterone levels (total as well as free testosterone) in humans and animals [1,2,6], and libido can increase in men [4–8] and women [9,10], and it is likely that any positive effect of fenugreek extracts is to a significant extent mediated via changes in androgen metabolism.

The benefits of extra supply of testosterone in small dosages are increased muscle mass, reduced visceral fat, and improved mood, cognitive function, and sexual function [7,8,11]. There are many people with low blood levels of testosterone who might have significantly reduced quality of life due to the low values [12].

Administration of high dosages of testosterone can cause a several serious side effects such as suppressed own production of testosterone, high blood pressure, increased beard growth, headaches, anxiety, depression, aggressiveness, and liver damage [13]. Thus, it is a need for supplements that might influence biological processes in a moderate way to enhance quality of life without too many side effects.

There are several different nutritional supplements intending to affect testosterone metabolism on the market, and Trigozim^R^ is one of them. The biologically active component of Trigozim^R^ is probably protodioscin [6], but the biological activity of Trigozim^R^ (and most other products on the market) is poorly documented in relation to testosterone metabolism.

The conventional way to monitor testosterone concentration in the body has been to measure total testosterone concentration in serum/plasma and estimate the free testosterone by also including the concentration of sex hormone binding globulin (SHBG) able to bind testosterone by high affinity [14]. It is assumed that free testosterone is the biologically active factor able to bind to the androgen receptor and thereby execute its physiological functions. Due to reports indicating that salivary testosterone is representative of the concentration of serum/plasma concentration of non-protein-bound free testosterone [15,16], we also measured testosterone in saliva.

Thus, the aim of our study was to investigate the effect of the food supplement Trigozim^R^, based on a special fenugreek herbal extract supplemented with several vitamins (the most important is probably vitamin D) and two minerals (zinc and magnesium), on the blood concentration of testosterone (free and bound), and self-reported libido in healthy men aged 40–80 years. C-reactive protein (CRP), alanine amino transferase (ALT) and creatinine were also measured in the blood as markers for possible tissue damage.

## Material and methods

### Participants

We recruited men from a database kept by Validator AS of approximately 70 000 subjects, of which 5 504 men where in the age of 40–80 years in the city of Oslo or its surroundings. 245 men out of 363 randomly asked (67% acceptance rate) accepted to participate. They were all evaluated to be healthy enough to participate in an intervention with 3 tablets of 1.2 g fenugreek extract per day for 12 weeks, and able to fill in a questionnaire concerning lifestyle and libido. When specific timepoints for booking of sampling was submitted 100 subjects showed up the first day, of which 95 men completed the intervention. The participants were instructed to keep their usual diet and alcohol consumption of less than one alcohol unit/day on average during the intervention. Blood and saliva samples were collected in the morning between 08.00 and 10.00. We started the final inclusion with signing of informed consent document March 6 and finished the recruitment March 14, 2024.

Subjects taking steroids or steroid-enhancing supplements or drugs were excluded as were men with high intake of alcohol or addictive drugs making them unable to adhere the experimental protocol. Serious psychiatric conditions were also a reason for exclusion to participate in the intervention.

Demographics will be descriptive and presented by randomised group.

### Design

**Fig 1:**
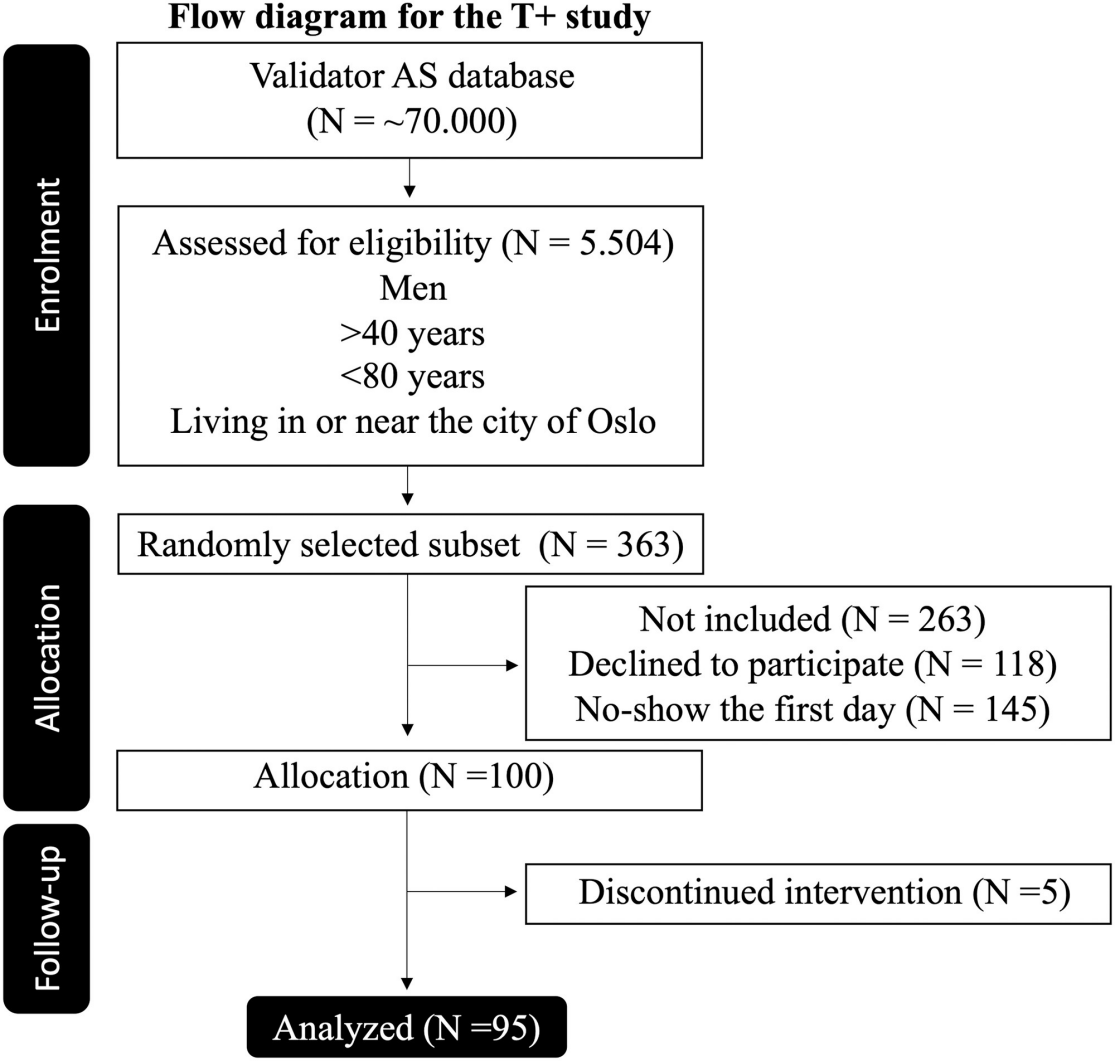
CONSORT flow diagram.

The intervention was double blind randomized with distribution of the participants in four groups (A-D) receiving three tablets Trigozim^R^/day containing 0, 600, 1200, or 1800 mg of fenugreek extract to be taken for 12 weeks with blood and saliva sampling at baseline (time 0), 2 weeks, 6 weeks, and 12 weeks after start of the intervention (Table 1). The randomization was performed by Rune Eilertsen (IPSOS/Validator) using the randomization function in Microsoft Excel, providing four groups (A, B, C, and D) getting 0–1800 mg of fenugreek extract as described below. All scientists and laboratory personnel were blinded for group allocation until all analyses were completed and the statistics were worked out. Please see Fig 1 for details.

**Table 1:** Content of one tablets Trigozim^R^ with 600 mg fenugreek extract. E341ii—dicalcium phosphate was used as excipient.

Ingredient	Dosage	RDA* (%)
Magnesium	60 mg	16
Zink	10 mg	100
Vitamin B1/thiamine	1,1 mg	100
Vitamin B2/riboflavin	1,4 mg	100
Vitamin B3/niacin/NE**	16 mg	100
Vitamin B5/pantothenic acid***	6 mg	100
Vitamin B6/pyridoxine	1,4 mg	100
Vitamin B7/biotin	50 μg	100
Vitamin B9/folic acid	200 μg	100
Vitamin B12	2,5 μg	100
Vitamin D3	10 μg	100

*RDA = recommended daily allowance.

**NE = niacin equivalents (1 mg niacin = 60 mg tryptophan).

***RDA not established.

*Group A* got no fenugreek extract in 3 tablets/day but with vitamins and minerals corresponding to 100% of recommended daily dosage, except for manganese (16%). *Group B* got 3 tablets Trigozim^R^ with 600 mg fenugreek extract in total, and with vitamins and minerals as in group A. *Group C* got 3 tablets Trigozim^R^ with 1200 mg fenugreek extract in total, and with vitamins and minerals as in group A. *Group D* got 3 tablets Trigozim^R^ with 1800 mg fenugreek extract in total, and with vitamins and minerals as in group A.

### Power calculation

The Trigozim^R^ intervention was powered to detect a significant change in plasma total/free testosterone concentration from baseline to 12 weeks, compared to placebo, as follows: Assuming based on previous data [2,4] that the treatment increase it by 10%-22% (Cohens *d* 0.28–0.67), and assuming 5% drop-out, we needed 48–76 participants given 80% likelihood of a statistically significant result with α = 0.05 using a two-way mixed ANOVA.

### Ethics

The study was approved by the Reginal Ethics Committee of South-East Norway, application number: 418176, and the study was carried out according to the Declaration of Helsinki–ethical principles for medical research of 2013 [17]. All participants signed a form including informed consent before entering the study.

### Analyses

Blood and saliva were collected in the morning between 08.00 and 10.00. Some blood was allowed to clot in sterile tubes and isolated by centrifugation at 4’C for 10 min with 1065 x G. Plasma was obtained after collection of blood in EDTA-containing vacutainer tubes. Saliva was collected in 15 mL Falcon^R^ tubes (product no 352097, Reynosa, Mexico) after rinsing the mouth with water and waiting for 10 min before the subjects were asked to think of lemon and collect the saliva. Testosterone in blood plasma and saliva was quantified after extraction with 2-propanol and analyzed by high performance liquid chromatography-mass spectrometry/mass spectrometry (HPLC-MS/MS). Testosterone is mostly bound to sex hormone binding globulin (SHBG) by high affinity and albumin, corticosterone binding globulin, and orosomucoid [14]. We have used a simplified algorithm: total serum concentration of testosterone (nmol/L) x 10/SHBG (nmol/L). SHBG was analyzed by enzyme linked immunosorbant assay (ELISA) kit from Euroimmune, Lübeck, Germany. 25hydroxy vitamin D_3_ (25OHvitD) was measured using HPLC-MS/MS. Testosterone, SHBG, and 25OHvitD were measured in the laboratory of Vitas Ltd, Oslo, Norway.

High sensitivity C-reactive protein (CRP) was monitored by immunotubimetry, creatinine was measured by an enzymatic colorimetric endpoint analysis, and alanine amino transferase (ALAT) was measured colorimetrically in serum using an autoanalyzer (Advia Chemistry XPT, Siemens Healthineers, Norway). Samples for zinc analysis were collected in Vacuette Li-heparin tubes with gel that migrates upwards during centrifugation, where it creates a stable barrier separating plasma from blood cells to prevent leakage of intracellular zinc into plasma. Plasma zinc concentrations were monitored by Inductively coupled plasma masspectrometry (ICP-MS) using Nexton 2000 ICP-MS (Perkin Elmer, Omaha, NE, USA). CRP, creatinine, ALAT, and zinc were all measured by Fürst Medical Laboratory (Oslo, Norway) as clinical blood biomarkers before as well as after 12 weeks of Trigozim^R^ intervention.

### Outcomes

The primary outcome was to measure the response in testosterone levels to fenugreek extract intake in both blood and saliva over 12 weeks. The secondary outcome was to evaluate safety in response to fenugreek extract intake.

### Adherance

Adherence was asessed by counting left over pills and measuring plasma concentration of 25OH vitamin D.

### Statistics

All data were analyzed using R v. 4.1.2. We modelled the data using repeated measures random intercept (for subjects) and random slope (for groups) mixed linear regression from the *lme4* R package. Data was analyzed in long format and missing data were handled by row wise deletion. There were no missing data on testosterone, but <1% missing data for safety measures (e.g. CRP, creatinine, ALAT and Zinc). Data were rank transformed to approximate normality prior to testing but plotted on the response scale for interpretability. Marginal means, confidence intervals and p-values were calculated using the *emmeans* R package. We corrected p-values for multiple testing using Tukey’s method. Plotting was performed using the *ggplot2* R package. Comparisons between and within groups regarding clinical characteristics and subjective experiences were performed using Wilcoxon ranked tests. The analyses were performed on an intention-to-treat basis, with per-protocol analyses included as supplementary data.

## Results

### Subjective questionnaire data

There were no differences in subject characteristics at baseline between the four different groups (Table 2). We detected no significant differences in subjective experiences between groups (Table 3), or in response to the intervention (Table 4). Trigozim^R^ supplementation resulted in weight loss after the intervention, with 1.1 kg weight loss observed in the group getting 1800 mg/d of Trigozim^R^ (Table 4) but in no other groups.

**Table 2:** Subject characteristics.

	Placebo (n = 22)		Trigozim^R^ (n = 73)		600 mg (n = 21)		1200 mg (n = 25)		1800 mg (n = 27)	
	Median	min, max	Median	min, max	Median	min, max	Median	min, max	Median	min, max
Age (years)	54	[40,68]	51	[40,75]	53	[40,75]	51	[40,72]	51	[40,72]
Height (meters)	181	[175,190]	180	[170,197]	181	[170,194]	178	[173,195]	180	[172,197]
Weight (kg)	85	[65,122]	85	[67,160]	85	[72,160]	80,5	[68,125]	87	[67,130]
Body mass index (kg/m2)	25,5	[21.2,35.6]	25,9	[20.8,42.5]	25,8	[21.4,42.5]	25,0	[20.8,36.1]	26,0	[21.3,33.8]
Stimulantia (1 = yes, 2 = no)	2	[1,2]	2	[1,2]	2	[1,2]	2	[1,2]	2	[1,2]
Tobacco (units per day)	0	[0,15]	0	[0,25]	0	[0,12]	0	[0,16]	0	[0,25]
Alcohol (units per day)	2	[0,12]	3	[0,14]	3	[0,14]	3	[0,10]	4	[0,14]

**Table 3:** Subjective experiences before and after 12 weeks intervention with Trigozim^R^ supplementation from 0–1800 mg extract/day.

	Placebo (n = 22)		Trigozim^R^ (n = 73)			600 mg (n = 21)			1200 mg (n = 25)			1800 mg (n = 27)		
**Baseline**	Median	min, max	Median	min, max	P	Median	min, max	P	Median	min, max	P	Median	min, max	P*
Erection	6	[4,7]	6	[2,7]	0,50	6	[3,7]	0,84	5	[2,7]	0,07	6	[3,7]	0,72
Fitness	5	[3,7]	5	[3,7]	0,68	5	[3,7]	0,67	5	[3,7]	0,19	5	[3,7]	0,89
Vitality	5	[4,7]	5	[2,7]	0,63	6	[2,7]	0,37	5	[2,7]	0,18	5	[3,7]	0,47
Sexual desire	5	[3,7]	5	[2,7]	0,79	6	[3,7]	0,60	5	[2,7]	0,83	5	[3,7]	0,34
Joy having sex	6	[3,7]	6	[3,7]	0,66	6	[3,7]	0,59	5	[3,7]	0,18	6	[3,7]	0,81
**After**														
Erection	6	[3,7]	6	[2,7]	0,75	6	[3,7]	0,73	6	[2,7]	0,60	6	[4,7]	0,34
Fitness	5	[4,7]	5	[2,7]	0,65	5	[2,7]	0,66	5	[3,7]	0,76	5	[4,7]	0,32
Vitality	6	[4,7]	5	[3,7]	0,59	6	[3,7]	0,62	5	[3,7]	0,29	5	[4,7]	0,47
Sexual desire	6	[4,7]	5	[3,7]	0,64	6	[4,7]	0,31	5	[3,7]	0,43	5	[3,7]	0,18
Joy having sex	6	[4,7]	6	[3,7]	0,35	6	[4,7]	0,66	6	[3,7]	0,09	6	[3,7]	0,34

*P values are vs. placebo using a Wilcoxon ranked test. The scores ranged from 0 (low) to 7 (high).

**Table 4:** Changes in body weight and subjective experiences after 12 weeks intervention with Trigozim^R^ (0–1800 mg/d).

	Placebo (n = 22)		P	Trigozim^R^ (n = 73)		P	600 mg (n = 21)		P	1200 mg (n = 25)		P	1800 mg (n = 27)		P
	Beta	95%CI		Beta	95%CI		Beta	95%CI		Beta	95%CI		Beta	95%CI	
Weight (kg)	-1.00	(-2.27,0.27)	0.105	-0.71	(-1.43,0.00)	0.046	0.01	(-0.96,0.98)	0.984	-0.98	(-2.77,0.82)	0.264	-1.14	(-1.81,-0.46)	0.001***
Erection (score)	0.01	(-0.25,0.27)	0.948	0.07	(-0.08,0.21)	0.354	0.05	(-0.20,0.30)	0.698	0.10	(-0.16,0.36)	0.430	0.05	(-0.19,0.30)	0.675
Fitness (score)	-0.01	(-0.28,0.26)	0.944	0.02	(-0.13,0.17)	0.808	-0.04	(-0.30,0.23)	0.782	0.04	(-0.23,0.31)	0.774	0.05	(-0.21,0.32)	0.689
Vitality (score)	0.02	(-0.24,0.28)	0.895	0.02	(-0.13,0.16)	0.817	0.02	(-0.24,0.27)	0.900	0.03	(-0.23,0.29)	0.845	0.01	(-0.25,0.27)	0.935
Sexual desire (score)	0.04	(-0.22,0.30)	0.741	0.03	(-0.11,0.18)	0.664	0.05	(-0.20,0.30)	0.686	0.01	(-0.24,0.26)	0.924	0.04	(-0.22,0.30)	0.788
Joy having sex (score)	0.06	(-0.19,0.31)	0.655	0.02	(-0.12,0.16)	0.794	0.05	(-0.20,0.29)	0.712	0.01	(-0.24,0.26)	0.916	0.00	(-0.25,0.24)	0.980

Data are beta with 95% confidence intervals and p-values from a Poission mixed model comparing after vs. before intervention within each group. Weight was analysed using a Gaussian mixed model. We observed no interaction effects between group and time.

### Objective data form blood and saliva

**Fig 2:**
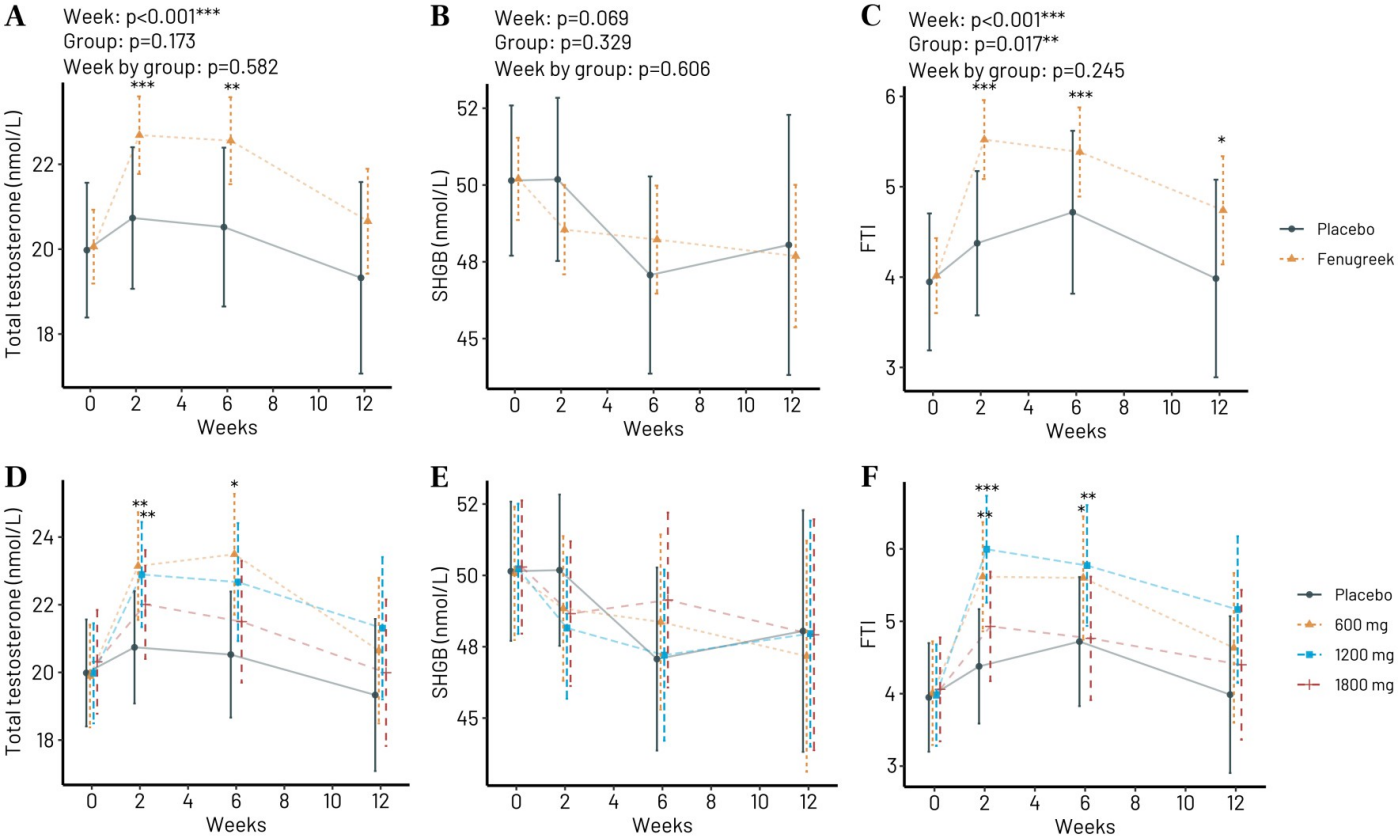
Blood responses to 0–12 weeks of Trigozim^R^ supplementation. The combined effect of any Trigozim^R^ dosage (0–1800 mg/d) on (A) total plasma testosterone concentration, (B) sex hormone binding globulin (SHGB), and (C) free testosterone index (FTI). Effect of different dosages of Trigozim^R^ vs. placebo on total testosterone (D), SHBG (E), and FTI (F). *p<0.05, **p<0.01 and ***p<0.001 vs. week 0 after correction for multiple testing by Tukeys method.

Considering all participants receiving any dosage of Trigozim^R^, there was significant increase in blood total testosterone concentrations at week 2 and 6 (Fig 2A), no change in blood SHBG concentrations, and significant increase in plasma free testosterone index (FTI) during the whole intervention period (Fig 2C). The observed changes with time were not significantly different from placebo (Fig 2A–2C). See S1 Table to S7 Table in S1 File for details.

By dividing the participants into different groups depending on the dosage of Trigozim^R^ (0–1800 mg/day), we observed significant differences at 2 and 6 weeks after start of the intervention for groups receiving 600 and 1200 mg/day of Trigozim^R^ (Fig 2D–2F). There were no differences in plasma concentrations of SHBG (Fig 2E). The observed changes with time were not significantly different from placebo (Fig 2D–2F). See S3 and S4 Table in S1 File for details.

**Fig 3:**
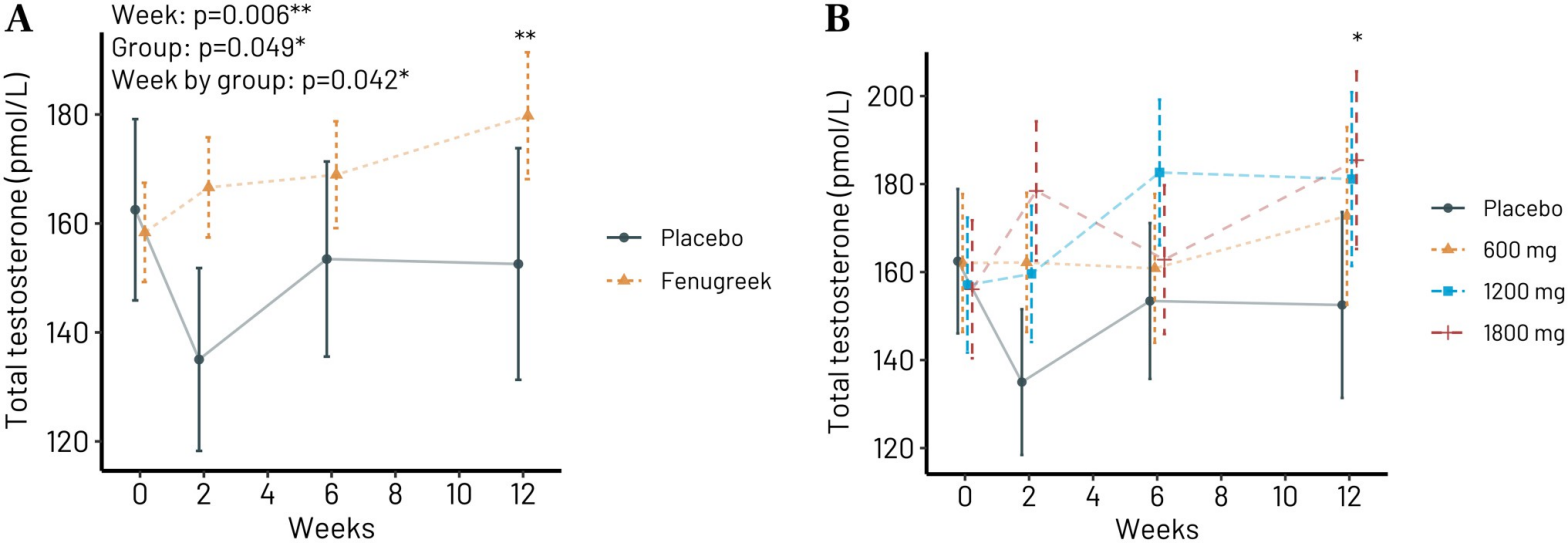
Saliva concentration of free testosterone after 0–12 weeks of Trigozim^R^ supplementation (0–1800 mg/d). (A) Trigozim^R^ vs. placebo; the combined effect of all Trigozim^R^ dosages. (B) Different dosages of Trigozim^R^ vs. placebo. *p<0.05 and **p<0.01 vs. week 0 after correction for multiple testing using Tukeys method.

Due to previous observations that testosterone in saliva is representative of the free testosterone concentration in blood plasma [18,19], we measured the total concentration of testosterone in saliva collected between 08.00 and 10.00 in the morning. Including all participants receiving any dosage of Trigozim^R^ we see from Fig 3A that there is significant increase in total testosterone also compared to placebo. Regarding individual dosages, there was only significant increase with 1800 mg/day (19%) of Trigozim^R^ (Fig 3B). See S5 and S6 Tables in S1 File for details.

Data in Figs 2 and 3 are calculated based on intention-to-treat, including participants on all three different dosages of Trigozim^R^ (600, 1200, and 1800 mg/day) irrespective of compliance concerning intake of tablets throughout the intervention period. If we considered the actual reported intake of tablets and only include participants with a compliance of ≥ 90%, we obtained similar results, but the significance was lost for FTI 0 to 12 week (S1 Fig), probably due to reduced statistical power because the samples size became smaller.

**Fig 4:**
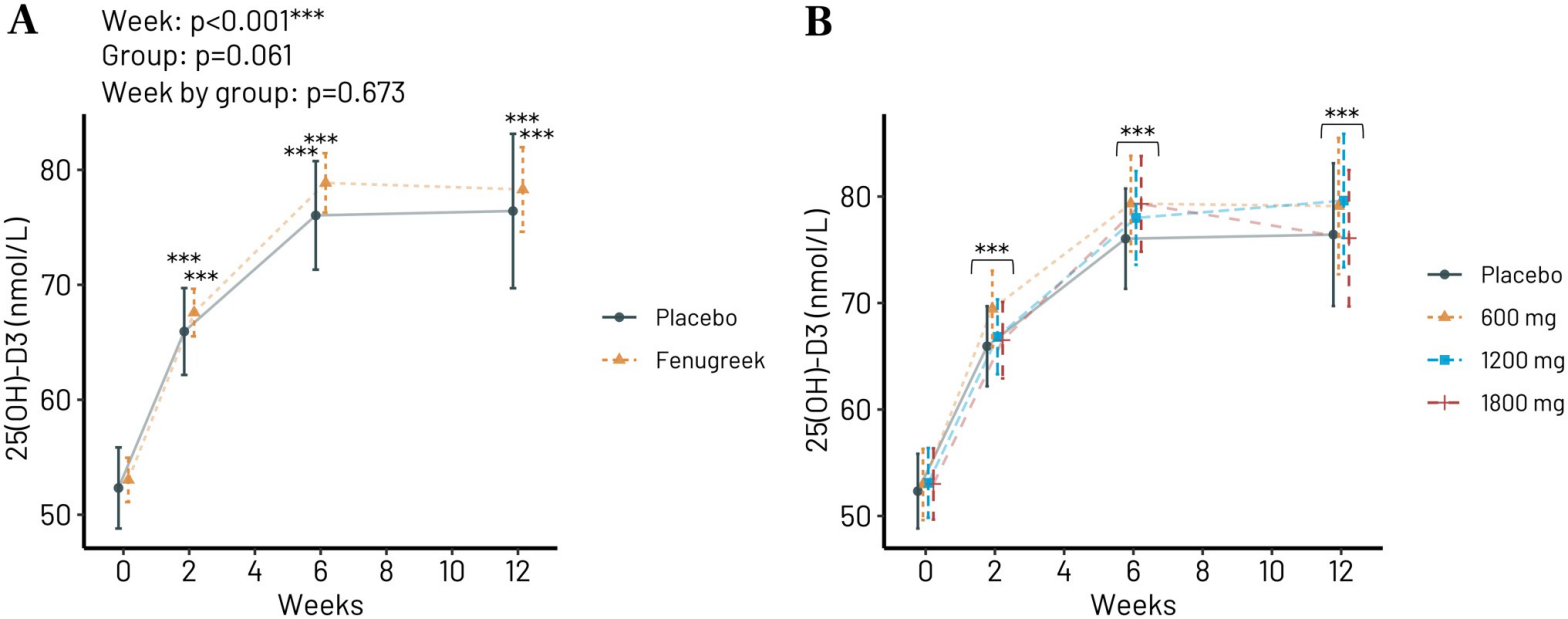
Plasma concentration of 25(OH) vitamin D_3_ after 0–12 weeks of Trigozim supplementation providing 30 ug/d of vitamin D in all groups. (A) The combined effect of any **Trigozim** dosage vs. placebo. (B) Different doses of **Trigozim** vs. placebo. ***p<0.001 vs. week 0 after correction for multiple testing using the Tukeys-method.

All participants receiving any type of Trigozim^R^ dosage experienced a significant increase in plasma concentration of 25OHvitD as expected because one tablet provided 10 ug of vitamin D (Fig 4), which is in accordance with previous observations [20].

**Fig 5:**
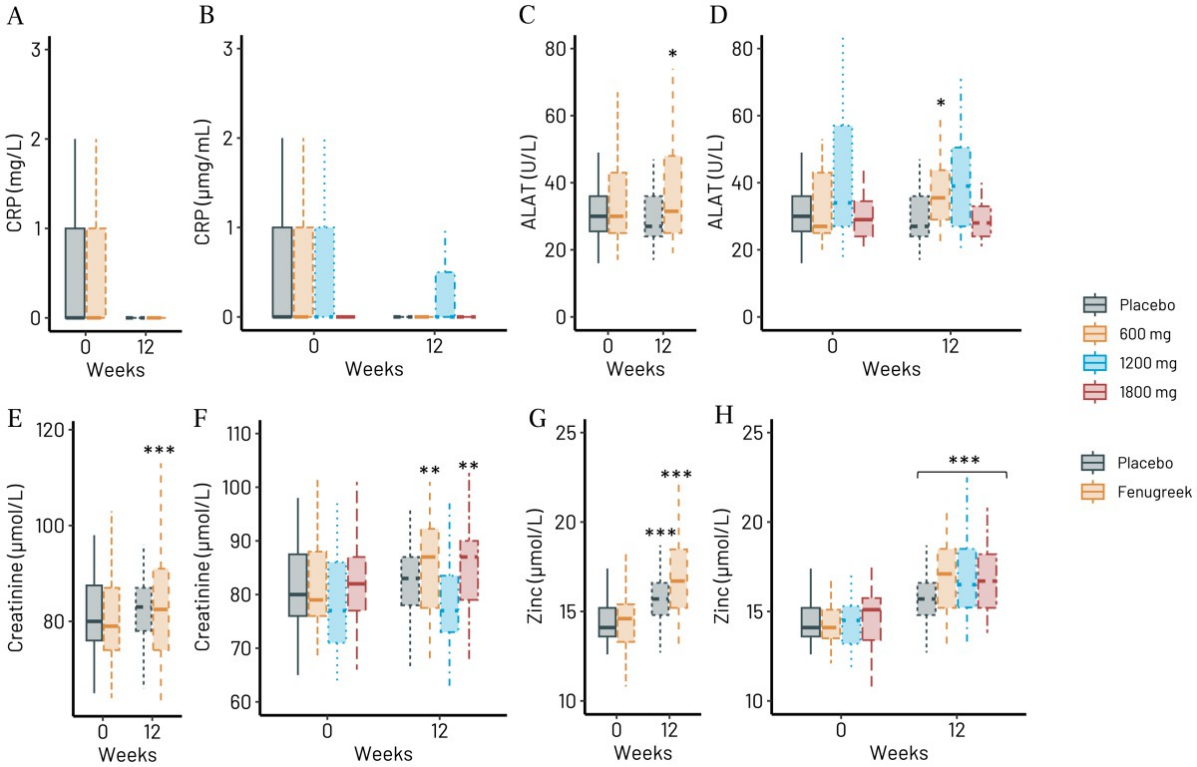
Clinical chemistry of serum/plasma before and after whole fenugreek group (600, 1200, and 1800 mg/day) and placebo. (A) hsC-reactive protein for all participants combined, (B) alanin aminotransferase, (C) creatinine, and (D) zinc; hsCRP for all subgroups, (F) ALAT, (G) creatinine, and (H) zinc. *p<0.05, **p<0.01 and ***p<0.001 vs. week 0 for each group.

Plasma concentrations of zinc increased significantly in all groups (Fig 5G and 5H) as expected with supplement of 10 mg of zinc provided in one tablet. The rational for having zinc in the Trigozim^R^ supplement is that zinc is essential for male fertility [21]. Zinc is the second most abundant trace element in humans and cannot be stored in the body; thus, regular dietary intake is required [22].

We observed no significant effect of Trigozim^R^ on concentrations of hs-CRP, ALAT, and creatinine as compared to placebo (Fig 5) but supplementation was associated with slightly and to some extent significantly increased serum concentrations of ALAT (Fig 5C and 5D) and creatinine (Fig 5E and 5F) during the 12 weeks intervention period compared to baseline for most groups. However, the enhanced levels were within the reference values also after the intervention. See S7 Table in S1 File for details.

## Discussion

95 of the included 100 men with average age in the early 50-ties, completed the study with no reported side effects but with a slight increase in serum concentrations of ALAT and creatinine.

The major finding in our present study is that there was significant effect of Trigozim^R^ on saliva concentration of total testosterone when all groups of men were included (Fig 2). There were also enhanced values of total testosterone concentration in serum and free testosterone index (FTI), although some time-points and dosages of TrigozimR did not show significant effects (Fig 1).

It has been a long-lasting discussion about which form of testosterone execute its biological effect. One model suggests that there is a low affinity binding of testosterone in blood to albumin, corticosterone binding globulin, and orosomucoid, whereas there is a high affinity binding to SHBG [14]. Moreover, it is assumed that the biologically active testosterone capable to bind to the androgen receptor, is the free testosterone (not protein bound). Several approaches and algorithms have been used [21]. One simple way to calculate free testosterone index (FTI) is total serum concentration of testosterone (nmol/L) x 10/SHBG (nmol/L) as we have used. The draw-back with this approach is that the algorithm depends on measuring both total testosterone and SHBG, thereby introducing inaccuracies in two measurements. Our FTI findings in serum are based on these measurements and may be hampered by measurement inaccuracies in two parameters (Fig 1).

Thus, we examined another option suggested by Vining et al. [23] that unconjugated steroids may reflect the concentration of free steroids in plasma. Our results indicate that measurements of testosterone in saliva may be more reliable than in serum (Figs 2 and 3). Saliva concentration of testosterone is similar to the data reported by Clifton et al. [19] in the same age group.

The relation between salivary content of testosterone and sexual behavior in both sexes has been studied by Macdowall et al. [23]. Among men, salivary testosterone was positively associated with both partnered sex (vaginal sex and concurrent partners) and masturbation, whereas among women, salivary testosterone was positively associated with masturbation. Thus, salivary testosterone levels seem to be related to functional aspects although we could not detect significant changes in subjective reporting in our relatively small population (91 men; Tables 3 and 4) as compared to more than 3600 subjects in Findings from the Third National Survey of Sexual Attitudes and Lifestyles (Natsal-3) [24] with considerably higher statistical power.

A possible explanation for the increase in total testosterone might be that the fenugreek extract may inhibit degradation of testosterone by inhibition of aromatase and 5 alpha-reductase [25] although this has not been tested directly with use of fenugreek.

A potential mechanism for the increase of free testosterone in blood or saliva might be that the plant steroid protodioscin may displace testosterone from their plasma binding proteins, although this has not been shown experimentally.

In addition to fenugreek extract the TrigozimR supplement includes 10 ug vitamin D_3_ and 10 mg zinc per three tablets (Table 1) corresponding to approximately 100% of RDA. Thus, the actual supplementation with vitamin D and zinc was similar to RDA. We observed consistent and significant increase for both these essential nutrients in all four groups of participants (Figs 3 and 5). This was expected because the provided amounts of vitamins and minerals were the same to all groups during the intervention. The plasma concentration of 25OHvitD seems to be quite optimal for the participants at this dosage, because there are several men and women in Norway with quite low blood levels of 25OHvitD [26].

The zinc concentration was higher than the reference value according to the Fürst laboratory [27] and Fallah et al. [22] but seems to represent acceptable values.

## Conclusions

The fenugreek extract named TrigozimR was safe and enhanced the plasma/serum concentrations of 25OHvitD and zinc as expected. There were no significant subjective alterations reported due to Trigozim^R^ intake, whereas there was increase in total plasma testosterone and FTI, and testosterone in saliva during the intervention. However, it was no significant difference in plasma concentration of testosterone compared to placebo. Because testosterone in saliva is only in its free form, analysis of testosterone in saliva may be the preferred way to monitor free testosterone in future studies. Sampling of saliva is quite simple with just rinsing of the mouth with tap water and waiting for 10 min before saliva is collected.

## Supporting information

S1 Fig Considering the real intake of TrigozimR supplements for 12 weeks intervention. (PDF)

S1 File Containing S1 to S7 Tables. (XLSX)

S2 File CONSORT checklist. (DOC)

S3 File (PDF)
