# Supplementary material for: Effect of a plant extract of fenugreek (Trigonella foenum-graecum) on testosterone in blood plasma and saliva in a double blind randomized controlled intervention study
Source: PLoS One. 2024 Sep 17;19(9):e0310170. doi: 10.1371/journal.pone.0310170 (PMC11407615; doi:10.1371/journal.pone.0310170)
Supplement: S1 Fig — (PDF) [file pone.0310170.s001.pdf]

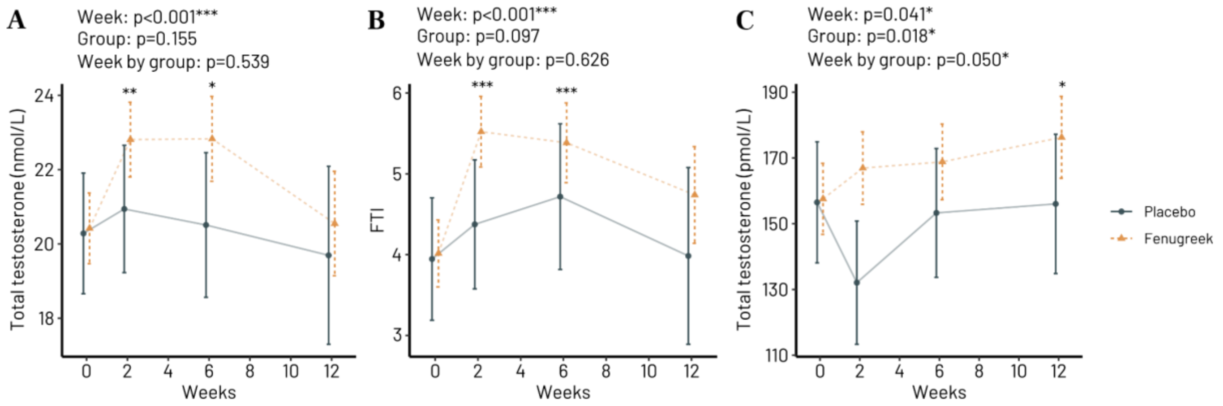

SFig. 1. Considering the real intake of TrigozimR supplements for 12 weeks intervention. The previously presented data (Figures 1-3) are calculated on basis of intention to treat (all participants took all tablets as instructed). If we perform the calculations based on only including participants who took $\geq$ 90% tablets we get total plasma testosterone concentration (A), and free testosterone index (FTI; (B), and saliva testosterone concentration (C).
