# Supplementary material for: Effect of a plant extract of fenugreek (Trigonella foenum-graecum) on testosterone in blood plasma and saliva in a double blind randomized controlled intervention study
Source: PLoS One. 2024 Sep 17;19(9):e0310170. doi: 10.1371/journal.pone.0310170 (PMC11407615; doi:10.1371/journal.pone.0310170)
Supplement: S3 File — (PDF) [file pone.0310170.s004.pdf]

Protocol Version 3 418176 T+ Study Protocol

Effect of T+ on Blood Concentrations of Free and Bound Testosterone

Background The active ingredient in the T+ supplement is believed to be the plant extract of fenugreek (*Trigonella foenum-graecum*), which has long been used to reduce sexual dysfunction such as impotence and to enhance libido. Fenugreek is one of the oldest and most used plant extracts in traditional medicine, containing compounds like steroids, alkaloids, saponins, polyphenols, flavonoids, various lipids, carbohydrates, amino acids, and hydrocarbons (1). A mixture of fenugreek plant components such as fruit, stem, and root has been used as spices, food, and as medicine/supplements for diabetes, high blood lipid levels, obesity, various cancers, inflammations, fungal and bacterial infections, and to enhance libido or anabolic effects in weight training (1-5). Recent studies indicate that some active components in fenugreek increase testosterone levels (total as well as free testosterone) in humans and animals (1, 2, 6), and libido may increase in men (4-8) and women (9-10), likely mediated significantly by changes in androgen metabolism. The benefits of additional small doses of testosterone include increased muscle mass, reduced visceral fat, and improved mood, cognitive function, and sexual function (7, 8, 11). Many individuals have low testosterone levels which could potentially lead to significantly reduced quality of life due to these low levels (12). High doses of testosterone can cause severe side effects such as suppressed natural production of testosterone, high blood pressure, increased beard growth, headaches, anxiety, depression, aggression, and liver damage (13).

Study Leader The study leader will be Professor MD, PhD, Christian A. Drevon (CAD) who has extensive experience with conducting intervention studies concerning nutrients, food items, and physical activity, as described in the attached CV and publication list. CAD is an emeritus at the University of Oslo (UiO) and works as a consultant for PurOmega AS. He is also a founder, board member, and consultant at the analysis laboratory Vitas AS in the Research Park in Oslo. Vitas will handle the analysis of blood samples in this intervention study.

Collaborating Enterprises To manage the recruitment of participants for the study, PurOmega Nordic AS has engaged Validator AS led by Rune Eilertsen. Validator AS is a company primarily assisting other market research companies with specialized tasks within sample handling and panel systems. Validator AS will be responsible for recruiting participants and collecting questionnaires; they have designed a sampling procedure and data handling procedure for use on Ipsos Norway's panel. Validator AS will be the research responsible institution and will sign a data processing agreement with Ipsos Norway AS. PurOmega Nordic AS will use the analysis laboratory Vitas AS in the Research Park, Gaustadalléen 21, 0349, Oslo (www.vitas.no) to take and analyze blood samples. According to the planned data collection procedures, Vitas AS will analyze, and report anonymized blood sample values that are solely identifiable via barcodes.

Project Plan **Purpose of the Project** We aim to measure the effect of various doses (1-3 tablets) at different times (0, 2, 6, and 12 weeks), for the intake of the supplement T+, produced by PurOmega Nordic AS. Blood and saliva samples will be taken in the morning between 8 and 10 am.

Materials & Methods The study will include 100 men aged 40-80 years who are healthy enough to complete a 12-week intervention study. After possible dropouts, we expect to have

20 men in each of four groups A) control group receiving placebo, while three groups of 20 men each will take B) 1, C) 2, or D) 3 doses of active ingredient T+ at 0, 2, 6, and 12 weeks. The study is conducted randomized and double-blind. The content of various ingredients is indicated in Table 1.

Table 1. Contents of various ingredients in a T+ tablet with 600 mg fenugreek extract

T+ Fenugreek/fenugreek extract 600 mg**
Magnesium 60 mg 16%*
Zinc 10 mg 100%*
Vitamin B1/thiamine 1.1 mg 100%*
Vitamin B2/riboflavin 1.4 mg 100%*
Vitamin B3/niacin/NE*** 16 mg 100%*
Vitamin B5/pantothenic acid 6 mg 100%**
Vitamin B6/pyridoxine 1.4 mg 100%*
Vitamin B7/biotin 50 µg 100%*
Vitamin B9/folic acid 200 ug 100%*
Vitamin B12 2,5 ug 100%* Vitamin D3 10 ug 100%*

* % recommended daily intake
** recommended daily intake not established
*** NE = niacin equivalents (1 mg niacin = 60 mg tryptophan)

The intervention is conducted while participants maintain their usual diet, and they must not have used medications or supplements that can affect testosterone metabolism. Additionally, participants must be mentally and physically capable of completing a 12-week intervention with three 1.2-gram tablets daily by filling out a digital questionnaire with relevant questions about lifestyle and health (appendix 1). The questionnaire will include information on age, diseases requiring medical supervision, use of medications, supplements, and narcotics to assess whether they can be included and will be capable of completing the intervention. We also ask about the subjective experience of sexual function before and after the intervention (appendix 1). At each time point, blood samples are taken through venipuncture to measure free and total testosterone, steroid hormone-binding globulin (SHBG), 25-hydroxy-vitamin D3, and zinc. Vitamin D3 and zinc are two of the ingredients in T+ that are specially added in the PurOmega Nordic AS T+ capsules. Therefore, these nutrients will also be analyzed in the blood samples. In addition, we will perform an analysis of free testosterone in saliva, which seems to be a good marker for free testosterone in the blood (14, 15). Saliva analyses for testosterone can be very attractive because they avoid the need for blood samples and seem to reflect a concentration of free testosterone similar to that in blood (15). The analyses are performed using various techniques such as High-Performance Liquid Chromatography (HPLC) mass spectrometry (MS), Enzyme Linked Immunosorbent Assay (ELISA), Gas Liquid Chromatography (GLC) mass spectrometry, and GLC Flame Ionization Chromatography (FID).

Handling of health information We collect very limited information on some dietary and drug habits. Moreover, we take four blood samples from all participants before (0) and after the intervention at 2, 6, and 12 weeks with the intake of three T+ supplement capsules with varying

amounts of active ingredients including the described minerals and vitamins. The placebo group (A) with 20 men will only receive three placebo tablets. Group B will receive three tablets with 1/3 active ingredients. Group C will receive three tablets with 2/3 active ingredients, while group D will receive three tablets with full active ingredients. The analyses are performed at Vitas AS, Research Park in Oslo.

Sources of human biological material As mentioned above, four blood samples will be taken through venipuncture. Some of the blood is dripped onto special filter cards to enable a more efficient analysis in the future. We will also use an advanced and very sensitive method directly on saliva to measure free testosterone. The test results are distributed to the research participants with assistance in interpreting the results. In addition, statistical calculations of the results will be done with a view to publication and publication of a scientific article.

Research ethical challenges There is minimal risk associated with the project, and all participants are informed in detail about how the study is conducted. Each participant and the group will learn about their personal results and the effect on the group of the intervention.

Funding Sources, Interests, and Dependencies PurOmega Nordic AS finances the entire study. IPSOS AS and Validator AS are paid for access to the database of men who have agreed to participate in medical studies. Vitas AS handles the blood sampling and analysis of the blood samples. The results are processed by Vitas AS in collaboration with project leader CAD. CAD is paid as a consultant by PurOmega. Each subject receives a Flax-lotto ticket worth 25 NOK after completing the study in addition to receiving the T+ supplement free for the 12-week intervention. There are no other financial relationships related to the research project.

Publication of Results The results will be processed in a report/article that will be published on the T+ website and/or issued in the form of printed material, possibly a scientific article. All other results and collected blood samples will be destroyed after the analyses are completed.

Literature

1. Venkata et al. A small plant with big benefits: Fenugreek (Trigonella foenum-graecum Linn.) for disease prevention and health promotion. Mol Nutr Food Res 2017, Jun 61, 6, doi: 10.1002/mnfr.201600950
2. Guo et al. Furosap, a novel fenugreek seed extract improves lean body mass and serum testosterone in a randomized, placebo-controlled, double-blind clinical investigation. Funct Foods Health Dis 2018, 8, 519-30
3. Wankhede et al. Beneficial effects of Fenugreek glycoside supplementation in male subjects during resistance training: A randomized controlled pilot study. *J Sport Health Science* 2016, 5, 176–82
4. Rao et al. Testofen, a specialised Trigonella foenum-graecum seed extract reduces age-related symptoms of androgen decrease, increases testosterone levels and improves sexual function in healthy aging males in a double-blind randomised clinical study. *Aging Male* 2016, 19, 134–42
5. Steels et al. Physiological aspects of male libido enhanced by standardized Trigonella foenum-graecum extract and mineral formulation. *Phytother Res* 2011, 25, 1294–300
6. Maheshwari et al. Efficacy of FurosapTM, a novel Trigonella foenum-graecum seed extract, in enhancing testosterone level and improving sperm profile in male volunteers. *Int J Med Sci* 2017, 14, 58-66

7. Miner M, Canty DJ, Shabsigh R. Testosterone replacement therapy in hypogonadal men: assessing benefits, risks, and best practices. *Postgrad Med* 2008, 120: 130–153
8. Beg S, Al-Khoury L, Cunningham GR. Testosterone replacement in men. *Curr Opin Endocrinol Diab Obes* 2008, 15: 364–370
9. Rao et al. Influence of a specialized Trigonella foenumgraecum seed extract (Libifem), on testosterone, estradiol and sexual function in healthy menstruating women, a randomised placebo-controlled study. *Phytother Res* 2015, 29, 1123–30
10. Palacios et al. Effect of a multi-ingredient-based food supplement on sexual function in women with low sexual desire. *BMC Women's Health* 2019, 19, 58 https://doi.org/10.1186/s12905-019-0755-9
11. Zhang XH, Filippi S, Morelli A et al. Testosterone restores diabetes-induced erectile dysfunction and sildenafil responsiveness in two distinct animal models of chemical diabetes. *J Sex Med* 2006, 3, 253–64
12. Mulligan T et al. Prevalence of hypogonadism in males aged at least 45 years: the HIM study. *Int J Clin Pract* 2006, 60, 762–9
13. Grech A, Breck J, Heidelbaugh J. Adverse effects of testosterone replacement therapy: an update on the evidence and controversy. *Ther Adv Drug Saf* 2014, 5, 190-200
14. Keveel et al. Distribution of salivary testosterone in men and women in a British general population-based sample: The third national survey of sexual attitudes and lifestyles (Natsal-3*). J Endocr Soc* 2017, 1, 14-25
15. Goncharov et al. Diagnostic significance of free salivary testosterone measurement using a direct luminescence immunoassay in healthy men and in patients with disorders of androgenic status. *Aging Male* 2006, 9:2, 111-22

17.10.2022 Christian A. Drevon
